# Population Genomics of Two Closely Related Anhydrobiotic Midges Reveals Differences in Adaptation to Extreme Desiccation

**DOI:** 10.1093/gbe/evad169

**Published:** 2023-09-14

**Authors:** Nurislam M Shaikhutdinov, Galya V Klink, Sofya K Garushyants, Olga S Kozlova, Alexander V Cherkasov, Takahiro Kikawada, Takashi Okuda, Dylo Pemba, Elena I Shagimardanova, Aleksey A Penin, Ruslan M Deviatiiarov, Guzel R Gazizova, Richard Cornette, Oleg A Gusev, Georgii A Bazykin

**Affiliations:** Regulatory Genomics Research Center, Institute of Fundamental Medicine and Biology, Kazan (Volga Region) Federal University, Kazan, Russia; Institute for Information Transmission Problems (Kharkevich Institute), Russian Academy of Sciences, Moscow, Russia; Institute for Information Transmission Problems (Kharkevich Institute), Russian Academy of Sciences, Moscow, Russia; Regulatory Genomics Research Center, Institute of Fundamental Medicine and Biology, Kazan (Volga Region) Federal University, Kazan, Russia; Regulatory Genomics Research Center, Institute of Fundamental Medicine and Biology, Kazan (Volga Region) Federal University, Kazan, Russia; National Agriculture and Food Research Organization (NARO), Institute of Agrobiological Sciences, Tsukuba, Ibaraki, Japan; Nemli project LLC, Tsuchiura, Ibaraki, Japan; Vectorborne Diseases Laboratory, University of Malawi Chancellor College, Zomba, Malawi; Regulatory Genomics Research Center, Institute of Fundamental Medicine and Biology, Kazan (Volga Region) Federal University, Kazan, Russia; Institute for Information Transmission Problems (Kharkevich Institute), Russian Academy of Sciences, Moscow, Russia; Regulatory Genomics Research Center, Institute of Fundamental Medicine and Biology, Kazan (Volga Region) Federal University, Kazan, Russia; Regulatory Genomics Research Center, Institute of Fundamental Medicine and Biology, Kazan (Volga Region) Federal University, Kazan, Russia; National Agriculture and Food Research Organization (NARO), Institute of Agrobiological Sciences, Tsukuba, Ibaraki, Japan; Regulatory Genomics Research Center, Institute of Fundamental Medicine and Biology, Kazan (Volga Region) Federal University, Kazan, Russia; Life Improvement by Future Technologies (LIFT) Center, Moscow, Russia; Intractable Disease Research Center, School of Medicine, Juntendo University, Tokyo, Japan; Institute for Information Transmission Problems (Kharkevich Institute), Russian Academy of Sciences, Moscow, Russia

**Keywords:** anhydrobiosis, paralogs, adaptive evolution, selective sweep, *P. vanderplanki*, *P*. *pembai*

## Abstract

The sleeping chironomid *Polypedilum vanderplanki* is capable of anhydrobiosis, a striking example of adaptation to extreme desiccation. Tolerance to complete desiccation in this species is associated with emergence of multiple paralogs of protective genes. One of the gene families highly expressed under anhydrobiosis and involved in this process is protein-L-isoaspartate (D-aspartate) O-methyltransferases (PIMTs). Recently, another closely related midge was discovered, *Polypedilum pembai*, which is able not only to tolerate desiccation but also to survive multiple desiccation–rehydration cycles. To investigate the evolution of anhydrobiosis in these species, we sequenced and assembled the genome of *P. pembai* and compared it with *P. vanderplanki* and also performed a population genomics analysis of several populations of *P. vanderplanki* and one population of *P. pembai.* We observe positive selection and radical changes in the genetic architecture of the *PIMT* locus between the two species, including its amplification in the *P. pembai* lineage. In particular, *PIMT-4*, the most highly expressed of these *PIMTs*, is present in six copies in the *P. pembai*; these copies differ in expression profiles, suggesting possible sub- or neofunctionalization. The nucleotide diversity of the genomic region carrying these new genes is decreased in *P. pembai,* but not in the orthologous region carrying the ancestral gene in *P. vanderplanki*, providing evidence for a selective sweep associated with postduplication adaptation in the former. Overall, our results suggest an extensive relatively recent and likely ongoing adaptation of the mechanisms of anhydrobiosis.

**Significance**

Tolerance to complete desiccation in *Polypedilum vanderplanki* is associated with the appearance of numerous paralogs of protective genes, but the evolution of these paralogs and their relationship with harsh environmental conditions has not been fully understood. One of the gene families highly expressed under anhydrobiosis and involved in this process in the midge are protein-L-isoaspartate (D-aspartate) O-methyltransferases (PIMTs). Using data from comparative genomics, transcriptomics, and population genomics, we found additional copies of *PIMT* paralogs that are under positive selection in the genome of a closely related anhydrobiotic midges from Malawi, *P. pembai*, which experiences more frequent desiccation–rehydration cycles due to differences in ecology of two species. These findings indicate ongoing adaptation of *P. pembai* to harsh environmental conditions and illustrate the importance of *PIMTs* in this adaptation. Such positive selection on paralogs of protective genes has probably driven also the adaptation of *P. vanderplanki* to its specific environment.

## Introduction

The anhydrobiotic midge *Polypedilum vanderplanki* (Chironomidae) (supplementary fig. S1*A*, Supplementary Material online) represents a unique example of adaptation to desiccation in its natural habitat. The larval stage of the midge inhabits temporary pools formed during rains on granite boulders in the northern part of Nigeria. The geographic range of *P. vanderplanki* maps to the semiarid regions of northern Nigeria, with some sparse observations in Burkina-Faso to the west and in Cameroon to the east (Okuda T, personal observations). The species was also described in Uganda (Hinton 1960). During the dry season, the larvae lose 99.2% of water, replacing it with trehalose combined with other molecular protectants. This allows the larva to survive the dry period in a dried state without detectable metabolism. After rehydration with the start of the rainy season, the larva returns to active life within less than an hour (Sogame and Kikawada 2017). Remarkably, the chironomids generally demonstrate high ecological plasticity, and their larvae are known for their ability to adapt to a wide range of extreme conditions, including high salinity, anaerobic environment, low pH, low or high temperatures, or desiccation (Armitage et al. 2012; Shaikhutdinov and Gusev 2022). For instance, another extremophilic midge *Belgica antarctica* that lives in the Antarctic can survive water loss of up to 70%, sustaining the low temperatures of the Antarctic climate (Kelley et al. 2014).

Physiological aspects of anhydrobiosis in the sleeping chironomid were investigated since the early 1950s (Hinton 1951), but studies of mechanisms underlying anhydrobiosis were launched only after the establishing of a successful rearing protocol for this species (Watanabe et al. 2002). Desiccation–rehydration cycles are accompanied by global accumulation of reactive oxygen species (ROS) in the cells (Cornette and Kikawada 2011). The survival rate of *P. vanderplanki* larvae was shown to decrease with successive desiccation–rehydration cycles due to the shortage of glycogen energy storage, and rehydration was proposed to be the most stressful stage due to the accumulation of oxidative and toxic compounds (Ryabova et al. 2020). Recent work on the molecular mechanisms underlying anhydrobiosis has led to identification of several groups of biological molecules that contribute to resistance to desiccation in *P. vanderplanki*. These include the protein-L-isoaspartate (D-aspartate) O-methyltransferases (PIMTs), proteins involved in trehalose biosynthesis, late embryogenesis proteins (LEA proteins), antioxidant system proteins, heat shock proteins, and DNA repair enzymes (Kikawada et al. 2006; Cornette and Kikawada 2011; Gusev et al. 2011). Comparison of *P. vanderplanki* to a nondesiccation-tolerant relative *P. nubifer* showed that the genes encoding desiccation-specific proteins (*PvLEA*, *PvPIMT*, thioredoxins [*PvTRX*], hemoglobins [*PvHb*]) in *P. vanderplanki* are present in multiple copies, highly transcribed and located in compact genomic clusters called Anhydrobiosis-Related gene Islands (ARIds) (Gusev et al. 2014).

One of the most interesting gene families associated with anhydrobiosis is *PIMT* genes, which have amplified to 14 copies in the *P. vanderplanki* genome. This group of genes is remarkable because those genes show one of the strongest changes in expression level in response to desiccation of the midge larva among all genes (Gusev et al. 2014); however, their molecular role in anhydrobiosis is not well understood (Deviatiiarov et al. 2017, 2020). The enzyme encoded by the ancestral *PIMT* belongs to the group of S-adenosylmethionine (SAM) dependent methyltransferases and catalyzes the repair of damaged amino acids such as L-isoaspartate and D-aspartate (Khare et al. 2011). Genes encoding these enzymes are highly conserved and are present in genomes of all eukaryotes (including insects), archaea, and gram-negative eubacteria in a single copy, with the exception of plants and some bacteria that have several *PIMT* isoforms (Desrosiers and Fanélus 2011). The activity of *PIMT* in animals is associated with resistance to stress factors and is directly related to life expectancy (Desrosiers and Fanélus 2011; Khare et al. 2011). It was shown that the accumulation of PIMT1 protein in *Arabidopsis thaliana* was associated with reduced accumulation of abnormal L-isoaspartyl residues and thus leads to increased longevity and vigor of dried seeds (Ogé et al. 2008). A family of *P. vanderplanki* genes, *PvPIMT*, is classified as *PIMTs* based on the presence of the conserved PIMT functional domain in the proteins encoded by them. However, individual *PvPIMTs* demonstrate marked differences in their amino- and carboxyterminal regions, suggesting that they may differ in their preferences for substrates, localization, or other specific properties, and not all of them show methyltransferase activity (Gusev et al. 2014).

While other lineages (tardigrades, rotifers, nematodes) have many anhydrobiotic organisms in their phyla, *P. vanderplanki* was until recently the only proven anhydrobiotic species both among the Chironomidae family and the whole Arthropoda phylum (Watanabe 2006). However, another midge from Malawi that was initially referred to as *P. vanderplanki* (McLachlan 1983a) is also able to survive in the desiccated state in the larval stage but differs in ecology from the Nigerian anhydrobiotic midge (Cornette et al. 2017). The geographic range of *P. pembai* is currently known only for the tropical savanna in the southern part of Malawi (McLachlan 1983b) with also a single report from Mozambique in the south (Okuda T, personal observation). While in Nigeria the dry season can last for up to 8 months without rain, in Malawi, it is interspersed with periodic sporadic rainfall (Cornette et al. 2017). Therefore, the larvae of the Malawian midge face several desiccation–rehydration cycles within one generation (Cornette et al. 2017). Together with morphological differences between the two chironomids, this allowed us to describe the Malawian midge as a new anhydrobiotic species *P. pembai* (Cornette et al. 2017) (supplementary fig. S1*B*, Supplementary Material online). Comparative analysis of genomic data from the two described anhydrobiotic species belonging to the same genus can help identify adaptations to desiccation at genome and transcriptome levels and understand how these species evolved the ability for anhydrobiosis.

In this study, we compare genomes of populations of *P. vanderplanki* and *P. pembai* to study the evolutionary adaptations to anhydrobiosis. We detect past duplication events leading to an increase in the number of desiccation response paralogs (*PIMTs*) in the lineage of *P. pembai* (*PpPIMTs*). Specifically, *PIMT4,* the paralog which is the most transcribed in response to desiccation among all *PIMTs* genes in both midges, is present in multiple copies in the genome of *P. pembai.* We find that the copies of *PpPIMT4* have experienced positive selection, suggesting that adaptation to anhydrobiosis is ongoing.

## Results

### Assembly and Characteristics of the Anhydrobiotic Midges Genomes

We obtained and sequenced seven populations of midges from two closely related species which diverged about 65–33 Mya (Cornette et al. 2017): *P. vanderplanki* and *P. pembai* (see Materials and Methods). The *P. vanderplanki* genome has been sequenced before (Gusev et al. 2014) and was reassembled recently utilizing Illumina, PacBio, and Hi-C sequencing data to obtain a chromosome-level genome (Yoshida et al. 2022). The current assembly represents a complete genome with four chromosomes. The size of the genome assembly is about 119 Mb with the GC content of 28.1%. The *P. pembai* genome has not been sequenced previously. Summary statistics for all analyzed samples are shown in supplementary tables S1 and S10, Supplementary Material online.

The draft genome of *P. pembai* was assembled de novo from a pooled sample of multiple individuals of *P. pembai*. About 57 million shotgun paired-end reads (100 bp) were used for the draft genome assembly of *P. pembai*. The estimated average coverage of the *P. pembai* draft genome is between 95× and 114×, and the final size of the draft genome assembly is ∼122 Mb. According to BUSCO, assembly completeness of *P. pembai* genome (by the same *Diptera* dataset) was estimated as 95.1% (93.6% for single-copy proteins), and 2.4% of the reference proteins were missing. The basic structural and functional features of *P. vanderplanki* and *P. pembai* genome assemblies are presented in supplementary table S2, Supplementary Material online. Although the *P. pembai* genome assembly has a lower quality than that of *P. vanderplanki*, the high completeness of the *P. pembai* assembly still permits comparative genomic analyses.

Additionally, we obtained high-throughput total RNA-seq data for *P. pembai* from midge larvae in the wet state and after desiccation for 24 and 48 h. Total RNA-seq data of *P. pembai* were used to compare transcription responses to the desiccation of *P. pembai* and *P. vanderplanki* larvae. The summary statistics for transcriptomics analysis are shown in supplementary table S3, Supplementary Material online.

### Population Genomics of *P. Vanderplanki*

**Fig. 1.**
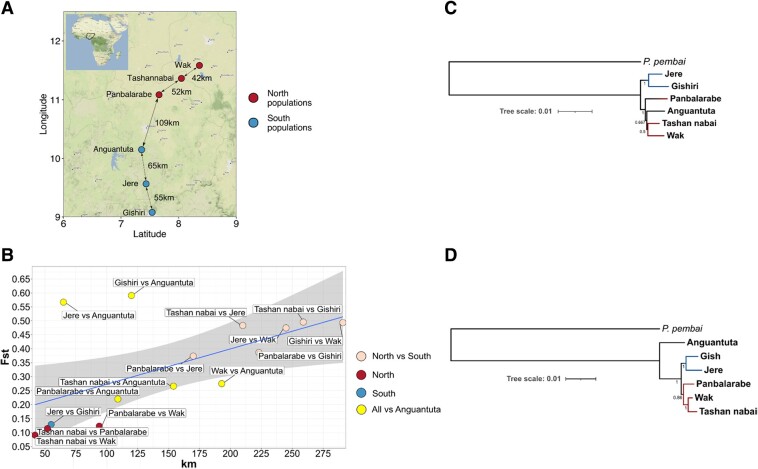
Geographic structure of *Polypedilum vanderplanki* populations. (*A*) Map of collection sites of *P. vanderplanki* populations. Map tiles by Stamen Design, under CC BY 4.0. Data by OpenStreetMap, under ODbL. The map was built using ggmap package (version 3.0.2) (Kahle and Wickham 2013). (*B*) Genetic distance as a function of geographic distance. (*C*) Rooted tree based on whole-genome consensus sequences for all Pv populations and Pp populations (Chikopa). (*D*) Rooted ML tree based on concatenated mtDNA protein-coding genes of all Pv populations and one Pp population (Chikopa). Values on branches represent support values—REQ values for (*C*); bootstrap values for (*D*). The 95% confidence band is shown.

We collected individuals of the adult stage of *P. vanderplanki* from six sampling sites in Nigeria (see Materials and Methods) (fig. 1*A*). Sampling sites were tens to hundreds of kilometers apart, which could limit gene flow between midge populations. Indeed, while the within-population nucleotide diversity (π) varied between 0.004 and 0.007 for different populations of *P. vanderplanki* (supplementary table S4 and fig. S2, Supplementary Material online), pairwise genetic distances between populations were higher: between 0.005 and 0.008 in the comparisons of northern populations, between 0.005 and 0.015 in the comparisons of southern populations, and between 0.009 and 0.014 in the north–south comparisons (supplementary table S4 and fig. S2, Supplementary Material online).

To better understand the genetic structure of the six considered populations, we analyzed the Wright fixation index (*Fst*). *Fst* was above-zero in all comparisons, varying between 0.114 and 0.591 (fig. 1*B*), indicating deviation from panmixis. It was lower, indicating weaker isolation, in the south–south and north–north comparisons (the smallest values in comparisons ranges between 0.09 and 0.13, with the exception for Jere vs. Anguantuta and Gishiri vs. Anguantuta comparisons, for which it equaled 0.56 and 0.59, respectively) than in the south–north comparisons (between 0.22 and 0.49). Consistently, *Fst* was correlated with the geographic distance between the analyzed populations (Mantel test R = 0.575; *P*-value = 0.0375), especially when the outlying Anguantuta population was excluded from analysis (Mantel test R = 0.954; *P*-value = 0.025) (fig. 1*B* and supplementary fig. S2, Supplementary Material online, respectively).

To confirm geographic subdivision of *P. vanderplanki* populations, we estimated phylogenetic distances based on consensus sequences obtained for each population for whole genomes (fig. 1*C*) and based on concatenated sets of mitochondrial protein-coding genes (fig. 1*D*). mtDNA phylogeny (fig. 1*D*) data provide a higher phylogenetic resolution, compared to the frequent approach of using just a single mtDNA gene such as COI (cytochrome c oxidase I) which often does not allow genetic differentiation of closely located populations (Havird and Santos 2014). The phylogenomic tree based on whole-genome approach clustered Anguantuta population within northern populations, contrasting with its geographical location and in line with its high genetic distance from southern populations (fig. 1*C*), although the tree based on mtDNA contained the northern and the southern clusters. Morphological analysis did not show significant differences between the populations, although the superior volsella of male genitalia, which is an important taxonomic feature, showed a lateral seta in 10–20% of the individuals from Wak, Tashan nabai, and Panbalarabe, whereas such seta was never observed in southern populations (supplementary table S5, Supplementary Material online). A slightly higher male antennal rate in the southern populations seemed also to support the genetic separation between northern and southern populations, including Anguantuta.

Based on the observed overall geographic subdivision of *P. vanderplanki* populations, we hypothesized that the mechanisms of anhydrobiosis encoded by the *PIMT* gene family could have evolved to adapt to their specific microenvironments. However, we found no support for this hypothesis either at the level of copy number variation or single-nucleotide variation of anhydrobiosis-related genes. Indeed, the coverage of individual *PIMT* paralogs involved in anhydrobiosis was similar between populations in our Pool-seq data, indicating that they were present in the same number of copies in different populations (supplementary table S6, Supplementary Material online). Similarly, the estimated πn/πs ratio of *PvPIMT* genes (supplementary table S7, Supplementary Material online) was low (πn/πs < 0.5) across all six *P. vanderplanki* populations, indicating the action of negative selection on all gene copies; we saw no evidence of relaxed negative selection or positive selection. Together, these findings indicate that the aspects of anhydrobiosis associated with *PvPIMT* genes are conserved within and between the populations of *P. vanderplanki*.

### Comparative Genomics of *P. vanderplanki* and *P. pembai*

While *P. vanderplanki* was the only anhydrobiotic insect known until recently, the discovery of another closely related species, *P. pembai,* changed this view (Cornette et al. 2017). *P. pembai* and *P. vanderplanki* are closely related according to the COI gene data (Cornette et al. 2017); however, morphological and cytological data (Cornette et al. 2017) indicate that they are two distinct species. Reproductive isolation is often associated with genetic distance above ∼10% (Mendelson et al. 2004; Elliot and Crespi 2006). Our analysis showed strong genetic differentiation between *P. vanderplanki* and *P. pembai* (*Fst* = 0.85 to 0.91, genetic distance varied between 0.077 and 0.08 depending on the considered *P. vanderplanki* population; supplementary table S8, Supplementary Material online), suggesting reproductive isolation between these species and supporting their species status. Within-population nucleotide diversity (π) for *P. pembai* equaled 0.004, similar to within-population nucleotide diversity (π) for *P. vanderplanki* populations.

Given the close relatedness of the two anhydrobiotic species, *P. vanderplanki* and *P. pembai* likely inherited anhydrobiosis from their common ancestor. Consistently with this assumption, we observe a similar genetic architecture of anhydrobiosis-associated genes, in particular, the presence of *LEA*, *LIL* (Lea-Island-Located), and *PIMT* multigene families which are absent in other insects. Still, due to the difference in the ecotopes of *P. pembai* and *P. vanderplanki*, the frequency of desiccation–rehydration cycles is expected to be higher for *P. pembai* (Cornette et al. 2017), suggesting the possibility of additional species-specific adaptation. Indeed, the genetic distance between *P. vanderplanki* and *P. pembai* was higher for chromosome 4 (10%) than for the other three chromosomes (7%) (supplementary table S8, Supplementary Material online). Chromosome 4 carries the majority of anhydrobiosis-related genes, including most *PIMTs* (*PvPIMT2* to *PvPIMT14*) in *P. vanderplanki,* so increased divergence may indicate accelerated evolution of genomic features connected with the ability to survive desiccation. We asked whether these differences have led to any observable differences in the genetics of anhydrobiosis between the two species.

### Adaptive Evolution of the *PIMT* Gene Family in *P. pembai*

**Fig. 2.**
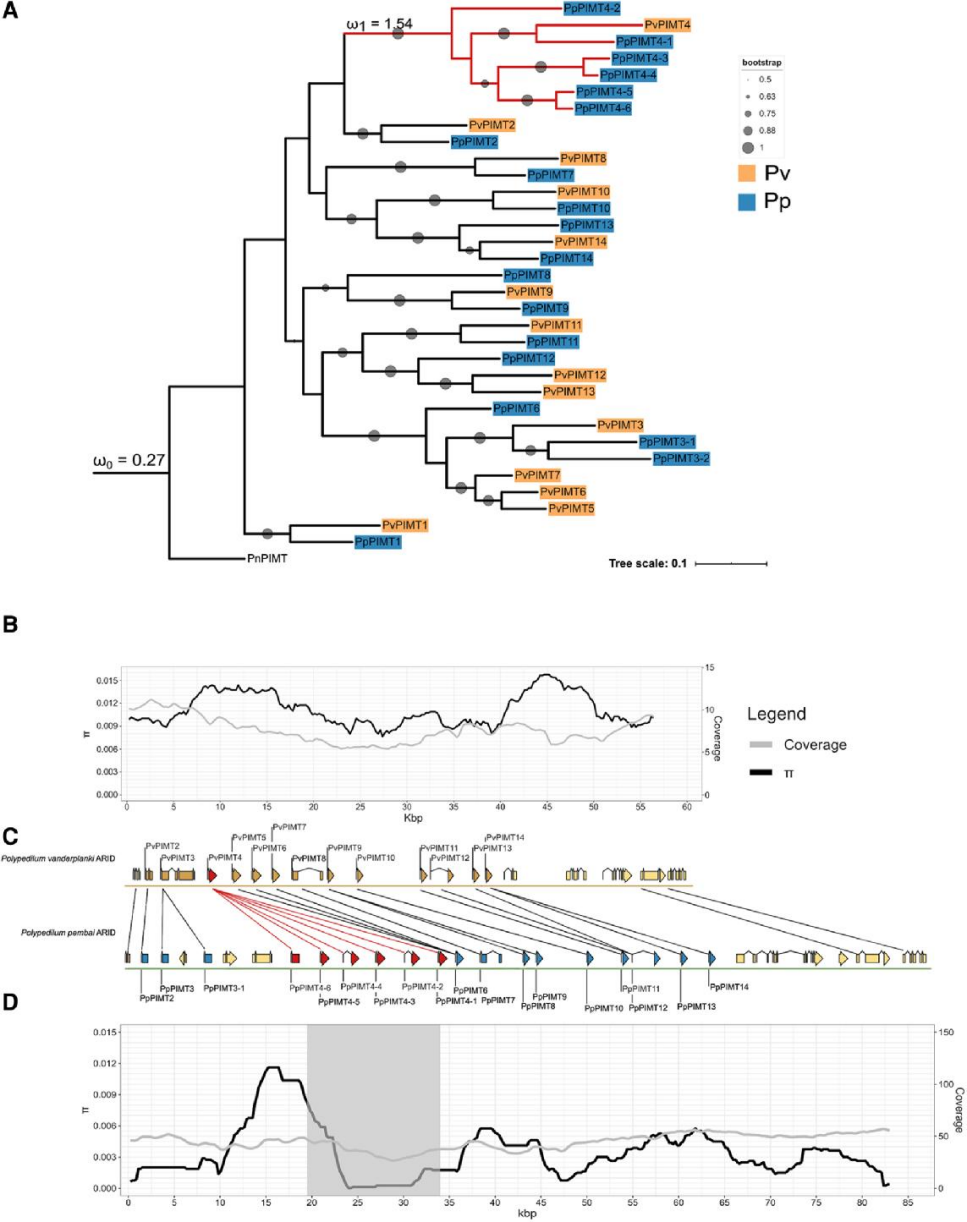
Comparative analysis of the *PIMT* ARId from two midges. (*A*) ML tree with bootstrap testing (100 replicates). Pv, *P. vanderplanki*; Pp, *P. pembai*, Pn, *P. nubifer*. Red color shows the clade under positive selection; blue color shows *P. pembai PIMTs*; orange color shows *P. vanderplanki PIMTs.* (*B*) Nucleotide diversity along *P. vanderplanki PIMT* ARId; (*C*) Comparison of gene order in the two ARId. Black lines represent homologous genes; (*D*) Nucleotide diversity along the *P. pembai PIMT* ARId. Gray color shows the zone of selective sweep found by Pool-hmm. Coverage curve shown in (*A, D*) is based on mapping of Tashan nabai population genomic data of *P. vanderplanki* and Chikopa population genomic data of *P. pembai.* ω_1_ stands for the foreground ratio, and ω_0_, for the background ratio.

Comparative genomic analysis revealed the presence of 14 paralogs of *PIMT* in *P. vanderplanki,* and 19 paralogs in *P. pembai* (fig. 2). To ensure that these differences do not result from assembly artifacts, we plot the read coverage along the *PIMT* locus for these two species (fig. 2*B–D*, gray lines). The coverage is uniform throughout the *PIMT* locus, without any evidence for differences which would have been expected under genome assembly errors. Together with the substantial divergence of *PpPIMT* paralogs from each other (fig. 2*A*), it is unlikely that the observed differences in numbers of paralogs result from assembly errors. Importantly, for other desiccation-related gene families, no gene amplification events were observed. On the contrary, *PvTRX*, *PvHb*, and *PvLEA* gene families have fewer genes in the *P. pembai* genome. While it is hard to prove gene loss given the incompleteness of genome assembly currently available for *P. pembai,* the observed absence of additional duplication events for those gene families make *PIMTs* unique.

**Fig. 3.**
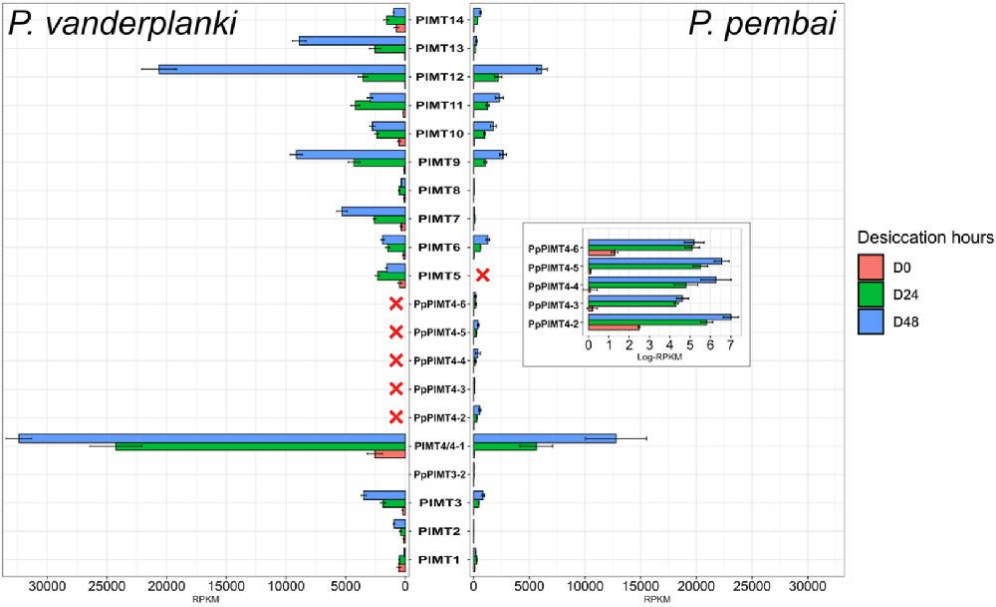
Differential expression of *PIMTs* in *P. vanderplanki* and *P. pembai* in response to desiccation. The expression profile of 14 *PvPIMTs* (left panel) and 19 *PpPIMTs* (right panel) during desiccation. The inset in the right panel represents the expression of duplicated paralogs in log scale. Red crosses identify paralogs absent in the corresponding genome. D0: control, D24, D48: desiccation for 24 and 48 h, respectively. Pv, *P. vanderplanki;* Pp, *P. pembai*.

Using transcriptomics data, we found that all 19 paralogs are differentially expressed in *P. pembai* in response to desiccation. The transcriptome profile of desiccation response in *PvPIMTs* and *PpPIMTs* is very similar between the two species. In particular, the two paralogs most highly expressed in response to desiccation in *P. pembai*, *PpPIMT4-1* (∼12,500 RPKM at D48 (desiccation for 48 h)) and *PpPIMT12,* are also the two most highly expressed paralogs in *P. vanderplanki* as *PvPIMT4* (>28,000 RPKM at D48) and *PvPIMT12* (∼6,000 RPKM at D48) (∼21,000 RRPKM at D48) (fig. 3). The expression rate of the other copies of *PIMT4* in *P. pembai, PpPIMT4-2* to *PpPIMT4-6,* remained low in response to desiccation but was still elevated till complete desiccation (D48). Overall, the transcriptional profile of *PIMT* was conserved between the two species (fig. 3), despite the fact that the two species diverged ∼49 Ma (Cornette et al. 2017).

Given the overall high conservation of the *PIMT* locus, we were interested in the difference in the number of *PIMT* copies between the two species. Phylogenetic analysis of the *PIMT* family indicates that this difference is due to a prolific clade of *P. pembai* paralogs in the *PIMT* gene family tree: one of the *P. vanderplanki* genes, *PIMT4*, is present in six copies, *PIMT4-1* to *PIMT4-6*, in *P. pembai* (fig. 2*A*). Analysis of genomic positions of *PIMTs* in the two species indicates that the order of genes is generally well-preserved between them; the increase in the size of the locus from 60 kb in *P. vanderplanki* to 85 kb in *P. pembai* is due to the presence of additional copies in *P. pembai* (fig. 2*C*). *PIMT4-1* to *PIMT4-6* are positioned in tandem in *P. pembai,* and appear in the same genomic neighborhood as *PIMT4* in *P. vanderplanki* (fig. 2*C*), supporting their homology.

The phylogenetic branches leading to four of these genes, *PIMT4-3* to *PIMT4-6*, are shorter than those corresponding to the *P. vanderplanki—P. pembai* divergence (fig. 2*A*), indicating that at least some of these differences in copy number between species were caused by a duplication in the *P. pembai* lineage rather than a gene loss in the *P. vanderplanki* lineage. To formally ask when this divergence occurred, we calibrated the phylogenetic tree using fossil data (Cornette et al. 2017), and used RelTime-ML model (Tamura et al. 2012) to date the duplication events. This analysis suggests that the amplification of this gene family has occurred in five duplication events, dating to 53.28, 51.82, 39.71, 8.89, and 7.26 Ma (supplementary fig. S3, Supplementary Material online). The earliest two of these events predate the *P. vanderplanki—P. pembai* divergence, indicating that subsequent evolution could have involved a gene loss in *P. vanderplanki;* conversely, the three later events have unambiguously occurred in the *P. pembai* lineage, indicating duplication in the *P. pembai* lineage.

We hypothesized that this amplification of the *PIMT* gene family has triggered adaptive accumulation of subsequent substitutions in the diverging copies. Consistent with this hypothesis, we observe a reduction in nucleotide diversity in the genomic region containing the additional paralogs of *PIMT4* in *P. pembai* (fig. 2*D*). This difference in diversity from neighboring regions is picked up by Pool-hmm (Boitard et al. 2013) as evidence for a past selective sweep in this region. No such reduction is observed in the homologous region of *P. vanderplanki* (fig. 2*B*), indicating that positive selection has only affected one of the two diverging species. Notably, this signature of selective sweep results from the postduplication evolution of the gene copies rather than the duplication events themselves.

**Fig. 4.**
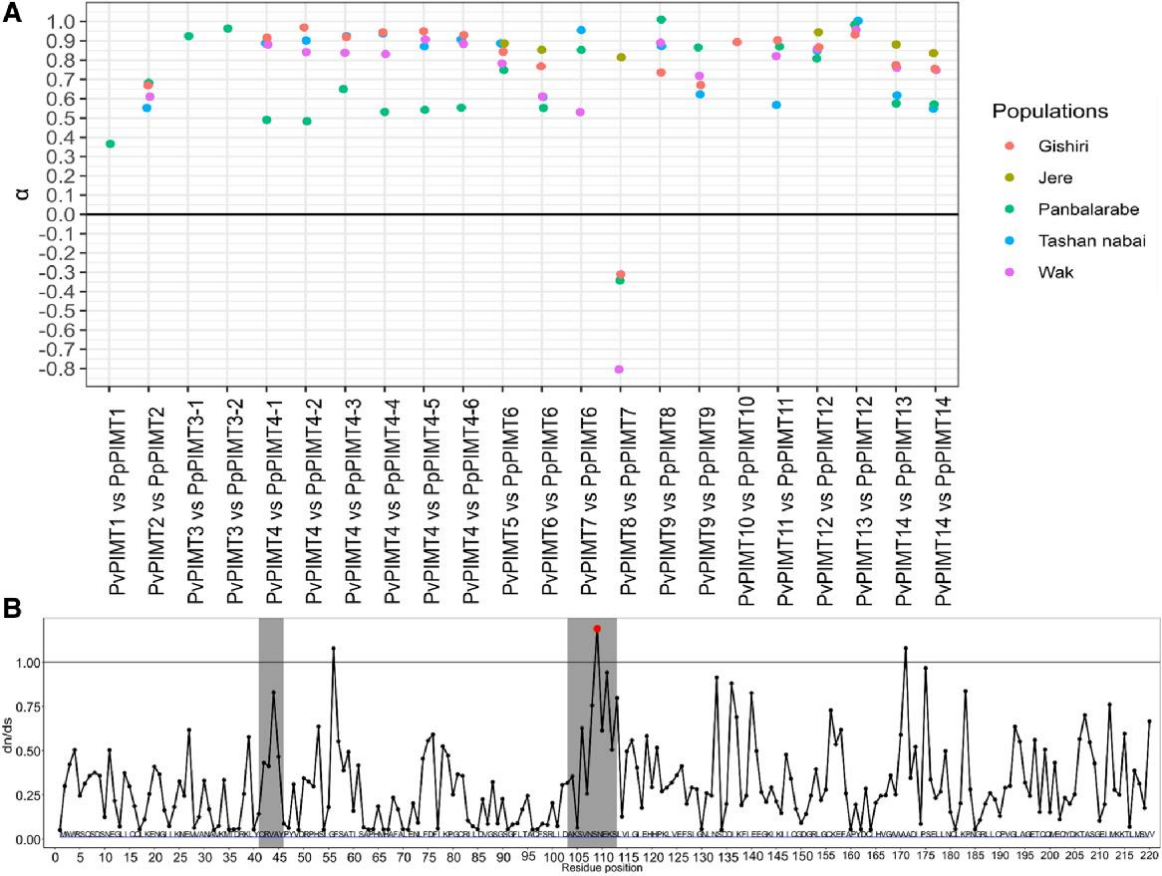
Selection at *PIMT* genes. (*A*) McDonald–Kreitman test of *PIMT* genes. McDonald–Kreitman test and α estimation (McDonald and Kreitman 1991; Smith and Eyre-Walker 2002) for each pair of closest PIMT orthologs between *P. vanderplanki* and *P. pembai,* calculated from *P. vanderplanki—P. pembai* divergence and *P. vanderplanki* polymorphism. Only those comparisons with at least 5 SNPs in the corresponding *P. vanderplanki* population are shown. (*B*) Identification of sites in *PIMTs* genes under positive selection. The only residue with BEB score >50 is shown in red. Gray boxes indicate the two short regions specific to the *PIMTs* of *Polypedilum* sp.

Given the observed trace of a selective sweep, we asked whether the *PIMT* gene family demonstrates evidence for positive selection on the encoded amino acid sequence. For this, we performed the McDonald–Kreitman test and estimated α, the fraction of advantageous nonsynonymous substitutions among those fixed between the two species (Smith and Eyre-Walker 2002), for each pair of closest *PIMT* orthologs between *P. vanderplanki* and *P. pembai* (fig. 4*A*, supplementary table S9, Supplementary Material online). For all but one comparison, we observe high values of α (mean α = 0.69), indicating strong positive selection acting on *PIMTs* after the divergence of the two species. α is lower for *PIMT1* and *PIMT2* than for the other paralogs, suggesting more prevalent negative selection maintaining the methyltransferase activity; and is the lowest for *PIMT8,* suggesting loss of function of this gene (consistent with its very weak expression, fig. 3).

To better understand the patterns of selection in *PIMT* genes, we asked how selection differs between protein sites. For this, we applied the site test for positive selection to the codon alignment of the *PIMT* gene family, using the M2a (selection) and M8 (beta & ω) models implemented in codeml (version 4.9). We found just one site with a statistically significant signal of positive selection (Bayes Empirical Bayes [BEB] score higher than 50%; fig. 4*B*). The sites with ω > 1 are positioned in two regions of the *PIMT* alignment: sites 41 to 46 and 103 to 113. These sites do not overlap the Drosophila *melanogaster* model of its only methyltransferase (Deviatiiarov et al. 2020).

Finally, given our observation of a selective sweep associated with the increase in the *PIMTs* copy number, we hypothesized that the subsequent evolution of the duplicated region could also be adaptive, in line with the neofunctionalization models (Innan and Kondrashov 2010). To test this, we used the branch test of codeml to compare the ω values in the clade that has expanded in the *P. pembai* lineage (foreground model, ω1) to those in the rest of the *PIMT* tree (background model, ω2). Strikingly, we observe a strong gene-wide signal of positive selection in the duplicated clade (ω1 = 1.5), while the rest of the tree evolves under strong negative selection (ω0 = 0.3; *P*-value < 0.001; fig. 2*A*). This indicates that the postduplication amino acid-changing mutations in the *PIMT* genes conferred a selective advantage.

## Discussion

The anhydrobiotic midges represent a unique example of adaptation to extreme conditions. Here, we provide the first comparative genomics analysis of two species of this group. By sequencing multiple populations of *P. vanderplanki,* we detect a high degree of genetic structure associated with geographic distance, indicating population subdivision. Such a geographic differentiation is perhaps unexpected for a flying insect and appears to be stronger than in other studied Diptera (Kumar and Singh 2017), despite the lack of visible or known geographic or ecological barriers between the studied populations. However, limited flight in *Polypedilum* sp. is consistent with other data. The maximal dispersal distance evaluated for *P. pembai* (formerly identified as *P. vanderplanki*) in Zomba, Malawi ranged between 0 and 446 month, suggesting a poor flight ability (McLachlan 1983a). In addition, many chironomid species stop swarming under windy conditions in order to avoid being blown away from their habitat (Armitage et al. 2012). Thus, dispersal of the anhydrobiotic midges with the winds on larger distances should be accidental, which corroborates isolation of the populations and explains the high Fst values observed here between Nigerian locations located over 50 km apart.

Geographic isolation creates ample opportunity for lineage-specific adaptation. According to fossil data, the heterogenous genus *Polypedilum*, grouping more than 520 described species inhabiting various environments in running and stagnant water, appeared around 75 Ma (Cranston et al. 2012) and most species do not survive desiccation for longer than a few days (Suemoto et al. 2004). However, a couple of species inhabiting temporary rock pools like *P. vanderplanki* can survive in dry mud for extended periods up to 6 months, but anhydrobiosis was not verified in these species, which may survive through a desiccation avoidance strategy (Cranston 2014; Cornette et al. 2022). True anhydrobiosis is expected to have been acquired exclusively in the common ancestor of *P. vanderplanki* and *P. pembai* between 65 and 33 Ma (Cornette et al. 2017). Our analysis indicates that these lineages accumulated ∼8% genetic distance, corresponding to the substitution rate of ∼10^−9^ per nucleotide per year which is consistent with other Dipterans (Keightley et al. 2014). While both these species are capable of anhydrobiosis, any potential differences in their ecology, such as recurrent cycles of desiccation–rehydration within a single dry season in the case of *P. pembai*, could lead to differences in evolutionary pathways of this system.

Most notably, we describe amplification of the gene family encoding an important protein involved in anhydrobiosis and provide evidence that genes of this family have accumulated adaptive amino acid substitutions after duplication. Specifically, we reveal the presence of positive selection acting on additional copies of methyltransferases in *P. pembai* (figs. 2*A* and 4*A*).

Gene duplication is one of the main sources of functional nucleotide diversity. It is believed that one of the main factors in formation of paralogs is adaptation to changing environmental conditions (Kondrashov 2012). For example, in the Antarctic notothenioid fish *Dissostichus mawsoni*, multiple duplications of the gene family of antifreeze glycoprotein (AFGP) occurred in response to the cold habitat of the fish based on the paralog of trypsinogen. It is noteworthy that duplication of the original trypsinogen and the appearance of the first antifreeze gene occurred 5–14 Ma, which roughly coincides with the time of mid-Miocene (10–14 Ma) when the Antarctic Ocean started to freeze (Chen et al. 1997). Frequently, amplification of gene families is associated with conditions of biotic or abiotic stress; for example, genes that have multiple copies in *Drosophila* were shown to be associated among others with pathogens defense and insecticide resistance (Rogers et al. 2015). In some instances, the beneficial effect of duplication seems to be associated with an increase in the dosage of the encoded gene; for example, the expression of duplicated genes in *Drosophila* is often increased more than two-fold in comparison with single-copy genes (Loehlin and Carrol 2016), suggesting that the high concentrations of the gene product are advantageous.

Here, we provide evidence that additional copies of *PIMT* genes in *P. pembai* genomes experienced adaptive evolution postduplication, possibly allowing the evolving *P. pembai* lineage to adapt to the changing environment. An increase in the copy number of *PIMT* from 1 to 14 copies from the last common ancestor of the *Polypedilum* genus, together with the fact that these additional copies do not show any S-adenosylmethionine-dependent methyltransferase activity (Deviatiiarov et al. 2020), suggests that these genes have acquired novel function(s). *PIMT1* and *PIMT2* from both species have low expression during anhydrobiosis, while other paralogs have increased expression (fold change D48 vs. D0 > 12 for *PvPIMT4*) in response to desiccation. It should be mentioned that *PvPIMT2* paralog shows less methyltransferase activity than *PvPIMT1* (Deviatiiarov et al. 2020), which partially explains the presence of weak differential expression between stages of anhydrobiosis (fig. 3). Currently, the exact biological role of *PIMT* paralogs during desiccation is not known; however, the evidence for selection acting on them and differential expression between desiccation time points (figs. 2–4) show that they play an important role. The positive selection observed in the McDonald–Kreitman analysis suggests continued adaptation to anhydrobiosis. The signal of positive selection is particularly strong in *PpPIMT4*, leading to an excess of nonsynonymous over synonymous substitutions in this entire clade, suggesting that *PpPIMT4* is particularly important in adaptation to extreme living conditions in *P. pembai*. Moreover, evidence for a recent selective sweep is observed in the *PIMT4* paralog region (22.5–35 kbp) which carries 4 out of 6 copies of *PIMT4*, further indicating that recent postduplication substitutions in this region were adaptive.

While our findings indicate that the postamplification evolution of the *PIMT* family in *P. pembai* has been largely adaptive, there is no direct evidence of the potential adaptive significance of the original amplification event itself. Still, the high rate of gene amplification suggests that it very likely could have been positively selected. Indeed, the *PIMT* gene has remained conserved and single copy over the course of billions of years, but has rapidly undergone five duplication events in just one lineage over the period of ∼50 Myr—a fact which appears to be hardly consistent with neutrality of these duplication events.

The mode and timing of positive selection in the process of gene amplification and subsequent evolution may help identify its cause. If indeed the amplification of *PIMT4* in *P. pembai* has been adaptive, this could be at least for two reasons: the beneficial effect of increased dosage due to an increased number of gene copies (Category II-a of Innan and Kondrashov 2010); or a beneficial modification of function of the paralogs at time of duplication (Category II-c of Innan and Kondrashov 2010).

The beneficial dosage effect model is favored by the fact that *PIMT* genes belong to stress genes, and the ancestral gene increases its expression under stress. Therefore, conceivably, the increase in the gene dosage caused by amplification could help cope with the stress conditions. However, under this scenario, no postduplication adaptive evolution is expected, because the function of the paralogs remains intact. Furthermore, a net increase of expression should be observed (Innan and Kondrashov 2010), that is the total expression of all paralogs in *P. pembai* should exceed the expression of the single ortholog in *P. vanderplanki*. This is not the case: while *PpPIMT4-1,* which is most similar to *PvPIMT4* (fig. 2*A*) and therefore probably carries the ancestral function, radically increases its expression in response to desiccation (fig. 3), no such increase is observed for its paralogs *PpPIMT4-2* to *PpPIMT4-6*. In a study of expression of human duplicates that arose since the human–macaque split (Lan and Pritchard 2016), it was shown that new paralogs tend to have a weaker expression than their ancestors. It was proposed by the authors of this study that down-regulation is a key first step enabling the initial survival of duplicates, followed by dosage sharing.

Finally, we note that other paralogs from the *PIMT* gene family in both species tend to have an at least ∼2× fold increase in expression rate between D24 and D48 desiccation points in *P. pembai*, suggesting that the D48 point is more stressful for the larvae and the cells need more mRNA and proteins of *PIMTs* paralogs at that point compared to D24. However, no major difference between D24 and D48 desiccation points in *P. pembai* is observed for *PpPIMT4-2* to *PpPIMT4-6* (fig. 3), suggesting that an increase in their dosage is not advantageous during increased desiccation stress.

Therefore, *PpPIMT4-2* to *PpPIMT4-6* are not consistent with the positive dosage effect model of gene-duplication evolution. The more plausible hypothesis is that the advantage is conferred by the divergence of the functions of these paralogs, perhaps due to differences in regulation or minor differences in sequence obtained at the time of duplication. Still, this question can be most confidently resolved by an experimental confirmation of the new functions of the *PpPIMT4* paralogs.

If the emergence of novel stress response genes of the *PIMT* family was not due to dosage effect as we suggest, why did they originate by duplication of the genes that already performed a similar function? Using *PIMT4* as an example, we put forward the following hypothesis: the genes involved in a response to a novel challenge are likely to originate from duplication of genes previously involved in a response to a similar challenge. The underlying logic is that although neofunctionalization is, by definition, associated with a change of function, this change is usually rather minor, and more easily obtained by modification of an existing similar function perhaps previously performed by a more promiscuous protein. Indeed, radical postduplication changes of function such as that observed in the arctic fish *D. mawsoni* are rare. Furthermore, even when the functions of paralogs diverge, they still typically continue to overlap, and an efficient stress response can be orchestrated by their joint expression: for example, the expression level of almost all genes of the *HSP70* group increases when heat shock stress is applied to the psammoreobiont chironomid *Orthoclaudiinae acuticauda* or applied a desiccation experiment to the desiccation-resistant *Paraborniella tonnoiri* (Kozlova et al. 2016).

If the two studied species, *P. vanderplankii* and *P. pembai,* differ in the architecture and the expression patterns of the *PIMT* locus, and these differences are adaptive as we show, what could underlie the distinct adaptations in these two species? We propose that it could be the differences in their ecology. In most cases, *P. vanderplanki* experiences just a single desiccation–rehydration cycle during its lifetime, meaning that there are only two time points when the larva is under the stress of ROS accumulation in the cell body: at time of desiccation and just after the start of rehydration. By contrast, *P. pembai* experiences multiple desiccation–rehydration cycles, meaning multiple ROS accumulations events and probably higher stress. Further supporting the additional burden of multiple desiccation–rehydration cycles, it was shown (Ryabova et al 2020) that each cycle depleted the trehalose and glycogen resources and that the deficit of these substrates prevented successful anhydrobiosis; consistently, the survival rate of *P. vanderplanki* larvae decreased with the number of such cycles. While repetitive cycles of desiccation–rehydration are facilitated by the high amount of glycogen and trehalose in *P. pembai* (Cornette et al. 2017), these resources are likely insufficient to induce successful anhydrobiosis. Conceivably, repair enzymes like *PIMTs* could counterbalance the higher oxidative stress damage experienced by *P. pembai* larvae. While it is yet impossible to elucidate the mechanism of such compensation, our data clearly indicate that with a similar toolkit, the strategies of desiccation tolerance in the two species are different.

Therefore, we expect that the increased stress experienced by *P. pembai* may lead to adaptive fixation of duplication events and postduplication substitutions in functionally important genes involved in anhydrobiosis (which includes a group of genes with high expression rates). We propose that the stress experienced by *P. pembai* larvae during desiccation and rehydration cycles is a much stronger selection pressure factor than that experienced by *P. vanderplanki*, leading to more rapid adaptation in the former. Measuring the fitness of the two species under different desiccation regimes, for example, by assessing the survival curves in one desiccation–rehydration cycle versus multiple cycles, requires further experimental studies.

When in the evolution of the two species did these differences emerge? The divergence level between paralogs can be affected by processes such as gene conversion or adaptive introgression, so our estimates of the timing of duplication events, which are based on divergence levels, should be taken with caution. Nevertheless, differences in divergence levels between paralogs imply that the amplification of the *PIMT* family in the *P. pembai* lineage has not occurred as a single event but instead has spanned a long period of time between ∼60 Ma and ∼5 Ma.

We suggest that the occurrence of paralogs in the *P. pembai* lineage coincided with the climate change towards aridity. Amplification events of *PIMTs* roughly coincide with a global climate event during late Paleocene epoch (ca. 55 Ma) called the Paleocene–Eocene Thermal Maximum that lasted for 50 ka, whereby the global temperature was increased by 8 °C on all continents. Also, the midge survived the warmest climate of the Cenozoic era in the Eocene epoch. After 33 Ma, the Oligocene was marked by general aridification in Africa, with, in particular, desertification of South Africa, and then during the early Miocene tropical rainforests were reduced, leaving a large proportion of the African continent covered with grassland and semiarid savannah (Feakins and Demenocal 2010). Actually, the time range of divergence between *P. vanderplanki* and *P. pembai* overlaps the Oligocene epoch (Cornette et al. 2017). Most likely, the accumulation of additional copies of methyltransferases in *P. pembai* can be explained by the climate differences between a less dry Malawi and a more arid Nigeria, which arose relatively recently. More frequent rain during the dry season in Malawi means that *P. pembai* larvae can experience multiple desiccation–rehydration cycles, in contrast to generally just one desiccation–rehydration cycle per dry season for *P. vanderplanki* larvae. Such a stress factor can both increase the negative selection maintaining the function of stress-tolerance genes, and lead to a spread of new beneficial mutations including gene duplications.

In summary, we show that while the genomes of the two anhydrobiotic midges, *P. vanderplanki* and *P. pembai,* carry the same set of gene families involved in anhydrobiosis, the composition of these families differs between the two species due to accumulated adaptive changes, and these genes are differentially expressed. While the molecular functions of the additional copies of *PIMTs* found in the *P. pembai* genome are presently unknown, the fact that they change their expression in response to desiccation implies that they are important for adaptation to anhydrobiosis. These additional copies of *PIMTs* are an example of ongoing or recent adaptation possibly associated with multiple desiccation–rehydration cycles per season encountered by *P. pembai*.

## Materials and Methods

### Material Collection and Genome Sequencing

We collected adult individuals (imago) of *P. vanderplanki* from temporary rock pools in the semiarid territories of the northern region of the Federal Republic of Nigeria. The sampling sites correspond to six locations: Tashan nabai, Wak, Panbalarabe, Jere, Gishiri, and Anguantuta (fig. 1*A*, supplementary table S11, Supplementary Material online). Names of samples (populations) match the names of villages where they were sampled. Samples of *P. pembai* were collected in the Southern region of the Republic of Malawi. Larvae were obtained from seasonal rock pools in Zomba city, in a location called Chikopa (15′ 23′ 422S, 35′ 18′ 877E) as described previously (Cornette et al. 2017). Desiccated larvae were brought back to Japan and stored in a desiccator until rehydration prior to use. Rehydrated larvae were reared on milk–agar diet and then developed to imago as described earlier (Watanabe et al. 2002). The imago were collected for the extraction of nucleic acids.

Genomic DNA was extracted from between 8 and 12 adult individuals from each population. Individuals from each population were homogenized together in Eppendorf plastic tubes (1.5 ml) with polypropylene pestle. Thus, each DNA pool was a collection of samples from a specific midge population. Highly pure genomic DNA extraction was performed with NucleoSpin Tissue kit (Clontech Takara), according to the instructions of the manufacturer. Genomic DNA concentration was estimated using a Qubit 3.0 fluorometer with Quantifluor dsDNA system (Promega).

Next, gDNA was fragmented using Covaris s220 (USA) DNA shearing protocol. The length of DNA fragments was estimated using Agilent Bioanalyzer 2100 (Agilent technologies). Libraries from each pool of gDNA were prepared using NEBNext Ultra II DNA Library Prep Kit for Illumina following the manufacturer's protocol. The concentration of libraries was measured by Qubit 3.0 fluorometer, its quality was verified on the Bioanalyzer using DNA High Sensitivity chip (Agilent technologies). Before sequencing, the number of molecules in each library was validated by real-time PCR using 2.5× reaction mixture for PCR-RV in the presence of EVA Green (Sintol, Russia) and primers for Illumina adapters (Evrogen, Russia). Further, taking into account the actual molar concentration of each library, they were diluted to 2 nM and pooled in accordance to the sequencing depth. The final pool was diluted to 11 pM.

Sequencing was carried out either on the HiSeq 2500 platform (Illumina) or HiSeq 2000 platform (Anguantuta population, *P. pembai*) in the pair-end mode using either HiSeq Rapid Pair-end Cluster Kit v2 and HiSeq Rapid SBS Kit v2 500 cycle kit (Illumina) reagents or TruSeq PE Cluster Kit v3 (Illumina) (Anguantuta population) and TruSeq RNA Library Prep Kit v2 (*P. pembai*). Agilent 2100 Bioanalyzer (Agilent Technologies, Santa Clara, CA) was used to obtain library lengths. Qubit 2.0 fluorometer and real-time PCR were used to quantify libraries. Length of forward and reverse reads in WGS libraries were either 250 bp for five *P. vanderplanki* samples or 100 bp for Anguantuta population and *P. pembai*. The mean length of insertion was 227 bp for Anguantuta population, 256 bp for *P. pembai* population and varied from 406 to 418 bp for the other five *P. vanderplanki* populations. Summary statistics for all populations including numbers of reads, average coverage, number of sites that pass filtering, genome assemblies quality metrics are shown in supplementary table S1, S2, and S10, Supplementary Material online.

RNA-seq data *of P. pembai* were obtained from wet larvae and larvae desiccated for 24 and 48 h. Several groups of 3–4 larvae of *P. pembai* were placed in a glass Petri dish (diameter 65 mm, height, 20 mm) on pieces of filter paper, filled with 0.44 ml of distilled water. Dishes were then immediately transferred to a desiccator (<5% relative humidity) at room temperature (24–26 °C), where water evaporated over a period of 48 h (0.22–0.23 ml day^−1^). Because *P. pembai* larvae represent field specimens and *P. vanderplanki* represents the inbred line, to minimize differences between the transcriptomic responses of both midges to desiccation, *P. pembai* larvae were reared in the laboratory for one month under conditions similar to those of *P. vanderplanki*.

*P. pembai* larvae from the Chikopa population in the wet, desiccated for 24 h or 48 h states were homogenized with polypropylene pestle in Eppendorf plastic tubes (1.5 ml) (8 to 10 larvae per sample) as described previously (Watanabe et al. 2002). For each state, we collected two replicas. Total RNA was extracted with Reliaprep RNA tissue Miniprep System (Promega), according to the manufacturer's instructions. Total RNA concentration was estimated using a Qubit 3.0 fluorometer with Quantifluor RNA system (Promega). First-strand cDNAs were synthesized from each sample. The libraries were validated by real-time PCR using 2.5× reaction mixture for RT-PCR with EVA Green (Synthol, Russia) and primers for Illumina adaptors (Eurogen, Russia). Then they were sequenced on a HiSeq 2500 platform (Illumina, USA) using the HiSeq PE Rapid Cluster Kit v2 and HiSeq Rapid SBS Kit v2 (200 cycles, Illumina, USA) in the 100 bp pair-end mode.

### Raw Data Processing, Assembly, Alignment and Annotation

FastQC (version 0.11.8) software was used as quality control of raw sequence data. Trimmomatic software (version 0.35) (Bolger et al. 2014) was used to cut adapter and other Illumina-specific sequences from the reads (TruSeq3-PE), using the following parameters: SLIDINGWINDOW:5:15.

For the genome assembly of *P. pembai*, we used MaSuRCA genome assembler (version 3.3.0) (Zimin et al. 2013) to create the set of genome scaffolds. Only those scaffolds >5,000 bp were retained in the final assembly. For the assembler, we set 500 bp as the mean and 50 bp as the standard deviation of the insert size. Quast and BUSCO tools (versions 4.6.3 and 3.0.2, respectively) (Gurevich et al. 2013; Waterhouse et al. 2018) were used for assembly quality assessment (supplementary table S2, Supplementary Material online). A tandem of RepeatModeler (version 1.0.11) and RepeatMasker (version 4.0.7) tools were used for identification of repeats and low-complexity regions, which were soft-masked for further gene prediction. This procedure was carried out with BRAKER tool (version 2.1.2) (Hoff et al. 2019) in ab initio mode. Functional annotation of corresponding protein models was performed using the InterProScan pipeline (version 5.26–65.0) (Jones et al. 2014).

Bwa-mem software (Li and Durbin 2009) was used to map the processed reads. As the reference *P. vanderlanki* genome, we used assembly version 5.0 (NCBI accession number: PRJNA660906). The reference *P. pembai* genome was obtained as part of this work and deposited to NCBI (accession number: PRJNA662005). To estimate the genetic distance between the two species, processed *P. pembai* reads were mapped to the *P. vanderlanki* reference genome assembly.

Samtools (version 1.9) (Li et al. 2009) and Picard software (version 2.20.0) were used to convert sam format with deduplication step (picard) to sorted bam files. Variant calling was performed with samtools mpileup (version 1.9) and bcftools call (or view) (version 1.9) options. Variant filtering was performed with GATK software (version 4.1.2.0); positions or variants with depth (DP) lower than 10 or mapping quality (QUAL) less than 20 were removed from vcf files.

### Phylogenetic Analysis, Polymorphisms Estimation, and Evolutionary Analysis

Phylogenetic reconstruction was performed using MEGA7 (Kumar et al. 2016) with maximum likelihood method and 100 bootstrap replicates. For this, we utilized the consensus gene sequences for each population. In such consensuses, all reference positions that have alternative alleles with frequencies greater than 0.5 were replaced with alternative alleles.

To perform phylogenetic reconstruction of populations, we used Pool-seq data (Tashan nabai, Panbalarabe, Wak, Jere, Gishiri, Anguantuta) that were separately mapped to Pv5.2 genome using with bwa mem (version 0.7.10-r789) (Li and Durbin 2009) with output converted into a sorted bam file with SAMtools (version 1.11-5-g0920974) (Li et al. 2009). Variant calling was performed with SAMtools mpileup and bcftools call (version 1.11-13-g78003de). Vcf files were filtered with GATK pipeline (version 4.1.19) with options “DP < 10 || QUAL < 20” (Li et al. 2009; Li 2011; Van der Auwera et al. 2013). Whole-genome consensus fasta files were made by bcftools consensus from each vcf files. Phylogenetic tree was built using alignment-free distance-based procedure by JolyTree software (version 2.1.211019ac) (Criscuolo 2019).

To date the duplication events of *PIMT* genes in both species, we used the RelTime-ML method included in MEGA7 software using known fossil data of Chironomidae family. We used the divergence date of *P. vanderpanki* and *P. pembai* as estimated by Cornette et al. (2017). In that paper, linearized maximum likelihood phylogenetic tree was inferred, showing a dated multigene phylogeny that was based on 18S and 28S ribosomal RNAs sequences, the mitochondrial COI protein-coding gene and the nuclear CAD protein-coding gene and was calibrated using data from (Cranston et al. 2012) for two phylogenetic nodes. Calibration was based on Culicimorpha fossil data that includes Chironomidae fossil data such as the most ancient definitive Chironomidae, *Aenne triassica,* from sedimentary rocks of the Rhaetian age (uppermost Triassic) limestone of England, dated as 202 Ma ± 1 Myr. For more information about fossil data used to calibrate the Chironomidae family, please refer to (Cranston et al. 2012) and (Cranston et al. 2010). Using that calibration, Cornette et al. (2017) put the divergence time between *P. vanderpanki* and *P. pembai* between 65 and 33 Ma; this date is based on several loci, both mitochondrial and nuclear, and therefore is presumably accurate.

To date the internal phylogenetic nodes, we reasoned that the time of species divergence matches that of divergence of single-copy orthologs. Thus, we used the common ancestor of *PpPIMT-1* and *PvPIMT-1* as calibration nodes. We then used the RelTime-ML model described in Tamura et al. (2012) to date the remaining internal nodes.

Importantly, our assessment of whether copy number changes involved gene loss or gene duplication is independent of the absolute dates; what matters is whether the LCA of the two copies predated or postdated the LCA of the two species.

Nucleotide diversity within and between populations (π) was measured with a custom script in R language (version 4.0.0) (Core Team R 2016). PoPoolation2 software tool (version 1.201) (Kofler et al. 2011) was used to measure allele frequencies and differences between populations and to estimate population differentiation summary statistics (Fst) along the genome with a sliding window approach. Fst was calculated only for positions with coverage not lower than 9 reads. Fst values were calculated (50 kbp windows, 25 kbp steps) from Pool-Seq data.

Orthogroups for *P. vanderplanki* and *P. pembai* were obtained by OrthoFinder (version 2.3.11) (Emms and Kelly 2019). Sequence alignment was performed by mafft (version 7.427) (Katoh and Standley 2013) and subsequent filtering by trimAL (version 1.4) software with default parameters (Capella-Gutiérrez et al. 2009). To calculate the d*n*/d*s* ratio, codeml program from paml package (version 4.9) (Yang 2007) was used with the following parameters: runmode = −2, seqtype = 1, CodonFreq = 0, model = 0, NSsites = 0, fix_kappa = 0, kappa = 2, fix_omega = 0, omega = 0.5; for branch model we used the following options: runmode = 0, model = 2, and model = 0; and for site model: runmode = 0, model = 0, NSsites = 0 1 2 3 7 8, omega = 1. To perform the McDonald–Kreitman test and α estimation (McDonald and Kreitman 1991; Smith and Eyre-Walker 2002), for each gene and population of *P. vanderplanki,* we compared the intrapopulation πn/πs with the pairwise interspecies d*n*/d*s*. d*n*/d*s* was calculated between the assembly (based on the Chikopa population) of *P. pembai* and the consensus (AF > 0.5) sequence of the corresponding population of *P. vanderplanki,* using the ape package (v.5.6-1) (Paradis and Schliep 2019) of Rstudio. The intrapopulation πn/πs of genes was measured with SNPGenie (version 1.0) (Nelson et al. 2015). Only genes with more than 5 SNPs in the corresponding population were used in the McDonald–Kreitman test.

### Analyses of Differential Expression at mRNA Level

Raw transcriptomic data for *P. vanderplanki* was obtained from genome browser “MidgeBase” (http://bertone.nises-f.affrc.go.jp/midgebase) and consisted of three experimental points (D0, D24, D48) with two replicates for each point. Extracted RNA was collected from whole larvae at 0, 24, and 48 h of dehydration (for each of the 50 individuals). Total RNA from four hydrated, dehydrating and rehydrated (*P. vanderplanki* only) larvae (for each of the 50 individuals) was extracted using Trizol (Life Technologies) and the RNeasy MiniKit (Qiagen, Hilden, Germany), according to the manufacturer's recommendations. TruSeq RNA Sample Preparation kit v.2 (Illumina) was used for preparation of RNA-seq libraries. Obtaining this dataset is described in more detail in the Gusev et al. (2014). This dataset was obtained using the same larvae desiccation procedure described above.

Reads were mapped using hisat2 (version 2.1.0) (Kim et al. 2019) to genome assemblies of *P. vanderplanki* and *P. pembai* accordingly. Raw count data were then used as input for the edgeR package (version 3.26.8) (Robinson et al. 2010) in R to analyze differentially expressed genes. Genes with less than 10 read counts were removed as a filtering step. To designate a gene as differentially expressed as a result of desiccation, we used a 2-fold change threshold.

## Supplementary Material

evad169_Supplementary_Data

## Data Availability

Data that support the findings of this study have been deposited in BioProject NCBI database under accession numbers PRJNA660906 (for *P. vanderplanki*) and PRJNA662005 (for *P. pembai*). Raw data of six *P. vanderplanki* and one *P. pembai* populations were deposited in the NCBI BioProject database under access numbers PRJNA996125 and PRJNA996593, respectively. All custom scripts used in that study can be found in github account—https://github.com/NurislamSheih.

## Supplementary material

Figures 1 to 3 and Tables 1 to 10 are available at *Genome Biology and Evolution* online (http://www.gbe.oxfordjournals.org/).
