# Supplementary material for: Population Genomics of Two Closely Related Anhydrobiotic Midges Reveals Differences in Adaptation to Extreme Desiccation
Source: Genome Biol Evol. 2023 Sep 14;15(10):evad169. doi: 10.1093/gbe/evad169 (PMC10558213; doi:10.1093/gbe/evad169)
Supplement: evad169_Supplementary_Data [file evad169_supplementary_data.docx]

**Supplemental Information for:**

**Population genomics of two closely related anhydrobiotic midges reveals differences in adaptation to extreme desiccation**

Nurislam M. Shaikhutdinov^*1,2^, Galya V. Klink^3^, Sofya K. Garushyants^3^, Olga S. Kozlova^2^, Alexander V. Cherkasov^1,2^, Takahiro Kikawada^4^, Takashi Okuda^5^, Dylo Pemba^6^, Elena I. Shagimardanova^2^, Aleksey A. Penin^3^, Ruslan M. Deviatiiarov^2^, Guzel R. Gazizova^2^, Richard Cornette^*4^, Oleg A. Gusev^*2,7,8^, Georgii A. Bazykin^*3^

^1^Center of Life Sciences, Skolkovo Institute of Science and Technology, Moscow, Russia

^2^Regulatory Genomics Research Center, Institute of Fundamental Medicine and Biology, Kazan (Volga region) Federal University, Kazan, Russia

^3^Institute for Information Transmission Problems (Kharkevich Institute), Russian Academy of Sciences, Moscow, Russia

^4^Institute of Agrobiological Sciences, National Agriculture and Food Research Organization (NARO), Tsukuba, Ibaraki, Japan.

^5^NEMLI PROJECT LLC, 2756 Okijuku, Tsuchiura, Ibaraki, Japan.

^6^University of Malawi. Chancellor College. Vectorborne Diseases Laboratory, Zomba, Malawi.

^7^RIKEN Cluster for Science, Technology and Innovation Hub, RIKEN, Yokohama, Kanagawa, Japan.

^8^RIKEN Center for Integrative Medical Sciences, RIKEN, Yokohama, Kanagawa, Japan.

***Corresponding authors:**

**E-mail:** [nurislam.shaikhutdinov@skoltech.ru](mailto:nurislam.shaikhutdinov@skoltech.ru), [oleg.gusev@riken.jp](mailto:oleg.gusev@riken.jp), [cornette@affrc.go.jp](mailto:cornette@affrc.go.jp), g[.bazykin@skoltech.ru](mailto:G.Bazykin@skoltech.ru)

**
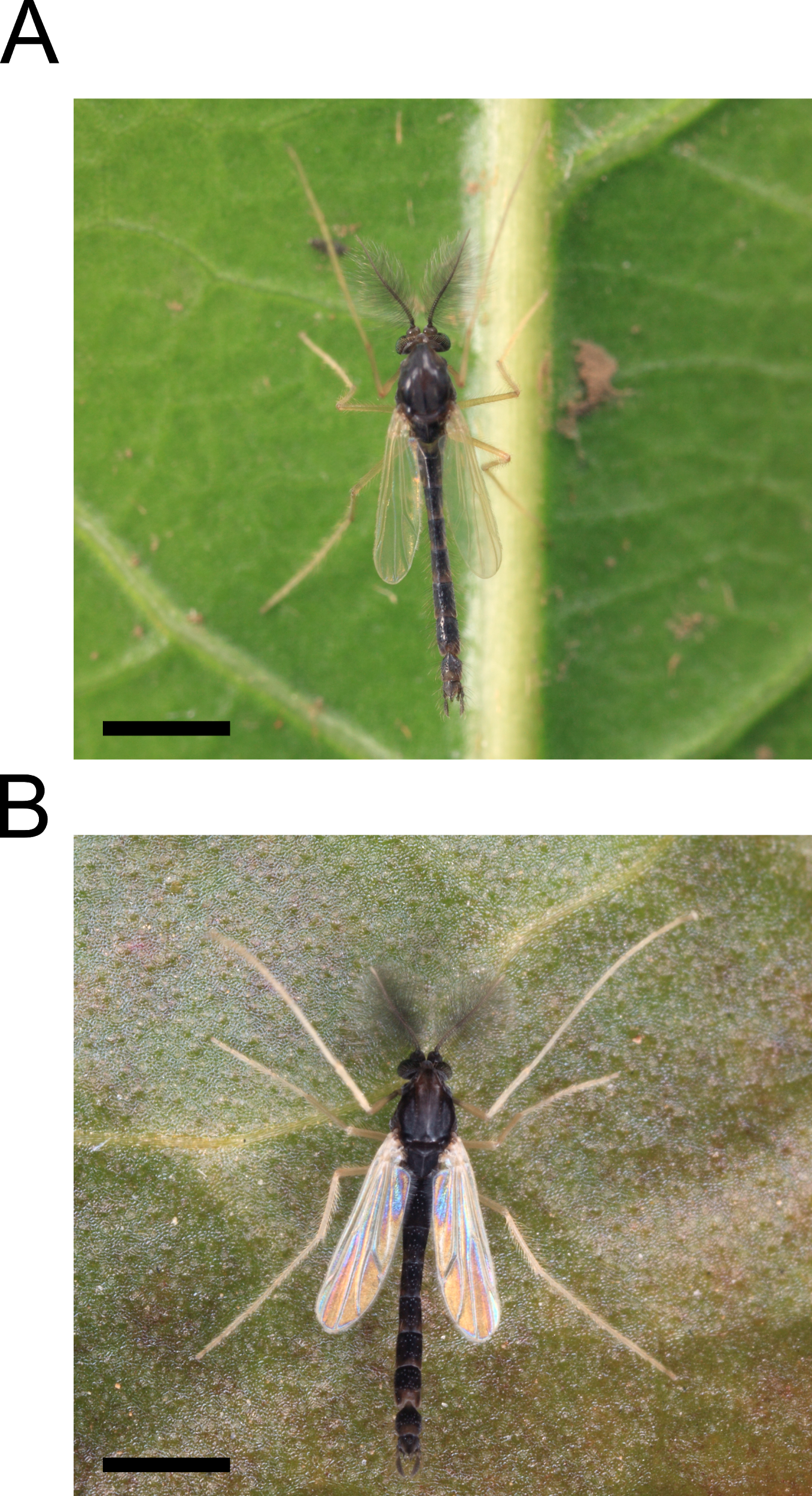
**

Fig. S1. Adult stages of *P. vanderplanki* (A) and *P. pembai* (B). Scale bars represent 1 mm.


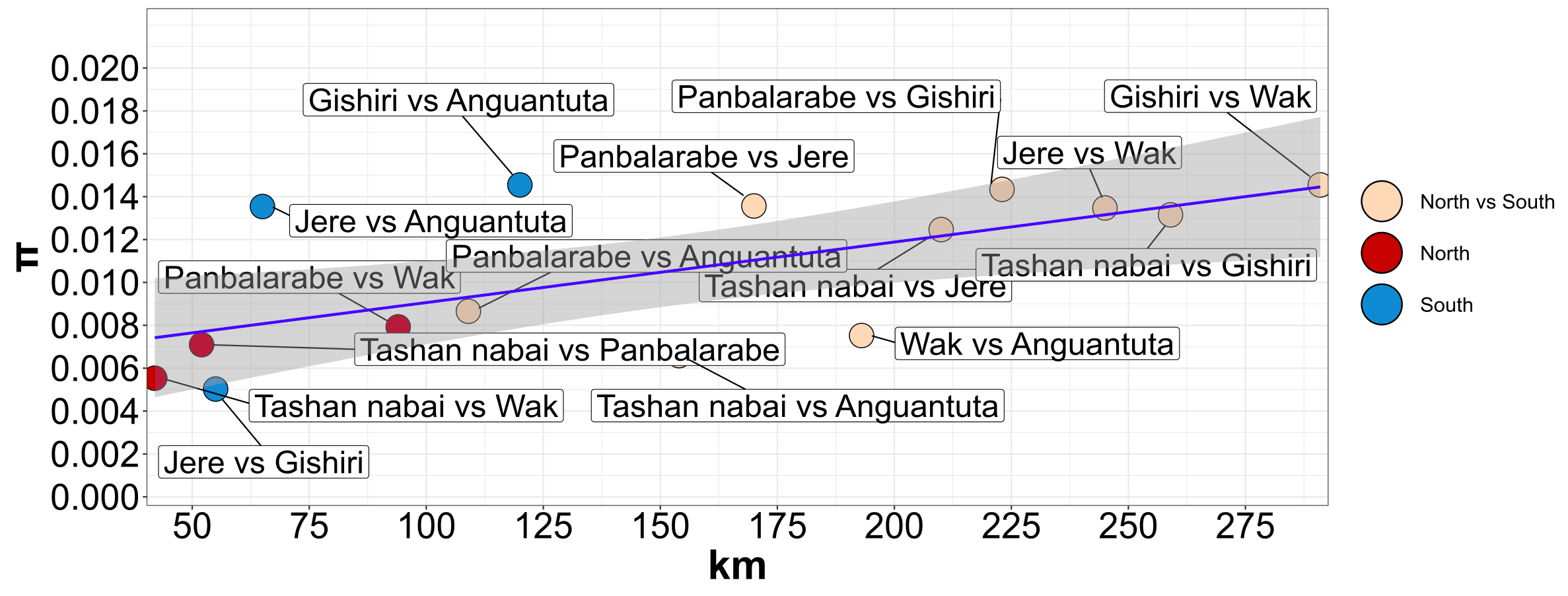


Fig. S2. Pairwise nucleotide diversity between all *P. vanderplanki* studied populations as a function of geographic distance (km). The grey color shows confidence intervals.


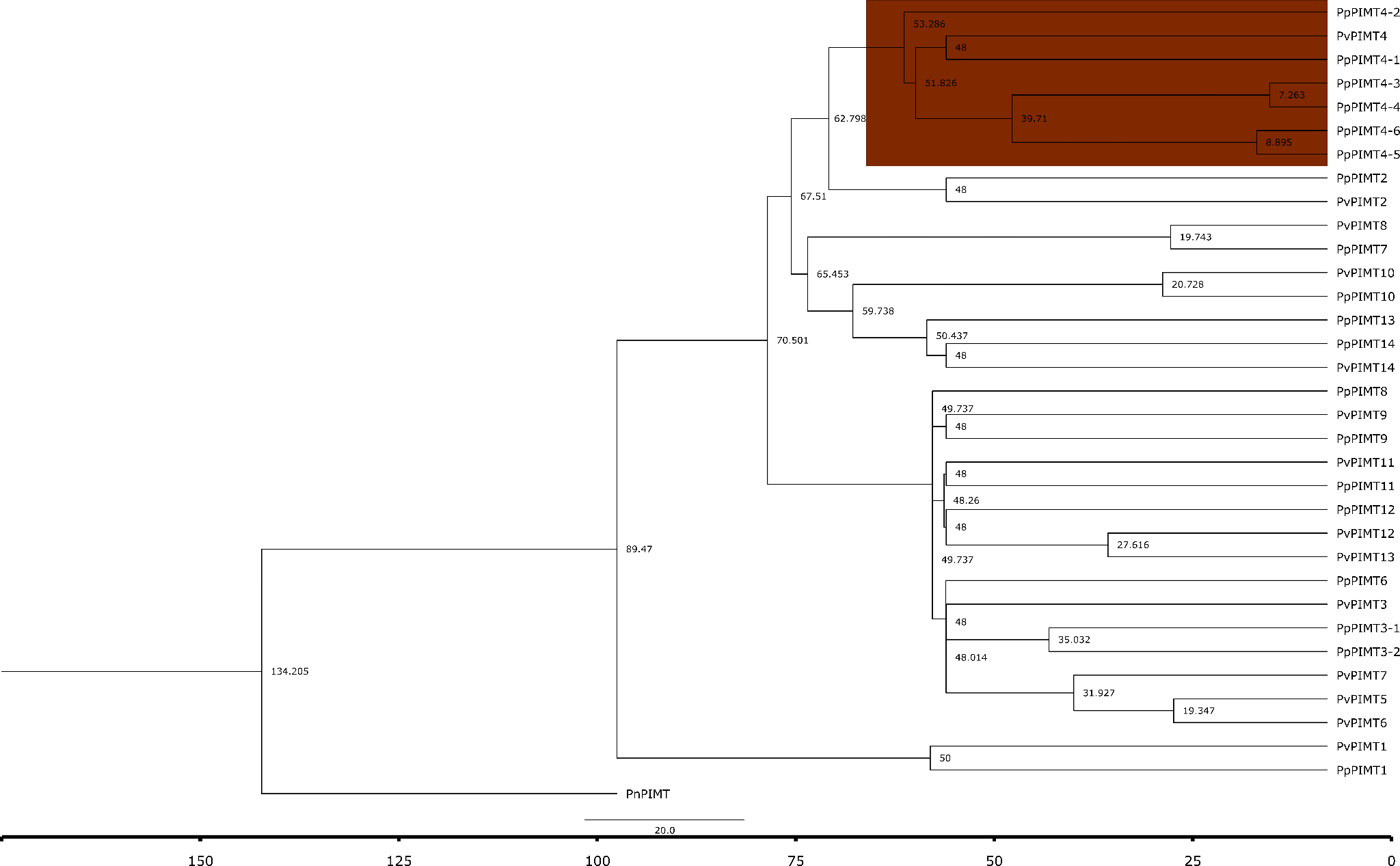


Fig. S3. Time divergence of *PIMT* paralogs using RelTime-ML method.

Table S1. Pool-seq data analysis summary

| Population | Total number of reads | Number of reads after trimming | Mapping  rate, % | Average coverage |
| --- | --- | --- | --- | --- |
| Tashan nabai | 6806884 | 6456690 | 98.87 | 12.03 |
| Panbalarabe | 8233398 | 7841488 | 98.91 | 14.29 |
| Wak | 9644900 | 9295736 | 98.76 | 17.16 |
| Jere | 6029944 | 5734942 | 98.01 | 10.23 |
| Gishiri | 8621858 | 8218478 | 98.81 | 14.98 |
| Anguantuta | 21303324 | 17243054 | 81.43 | 10.34 |
| Сhikopa | 114356234 | 100472154 | 90.1 | 163.5 |

Table S2. The statistics of the assembled genomes of *P. vanderplanki* and *P. pembai.*

|  | *P. pembai* | *P. vanderplanki* |
| --- | --- | --- |
| Number of scaffolds | 4662 | 4 |
| Genome size, Mbp | 122 | 119 |
| Coverage | ~110X | ~450X |
| N50 (scaffold) | 40.9 kbp | 34 Mbp |
| The amount of N per 100 kbp | 208.78 | 1699.08 |
| BUSCO* | C: 95.1%;  D:1.5%;  F: 2.5%;  M: 2.4% | C: 96.8%;  D: 0.9%;  F: 1.7%;  M: 1.5% |
| Genomic G+C content, % | 28.6 | 28.1 |
| Predicted numbers of protein-coding genes | 15068 | 17852 |
| Percentage of genes in orthogroups | 93.8 | 91.9 |
| Genes common for both species (number of 1:1 orthologues) | 10625 | 10625 |

Note. —Letters in BUSCO correspond to complete, duplicated, fragmented and missing.

Table S3. Total RNA-seq summary characteristics for *P. pembai.*

| Sample | Total number of reads (2x100) | Overall alignment rate, % | Uniquely mapped reads, % | Multi mapped reads, % |
| --- | --- | --- | --- | --- |
| D0 (first replica) | 133996878 | 19.31 | 11.13 | 3.63 |
| D0 (second replica) | 124822970 | 17.33 | 10.19 | 2.89 |
| D24 (first replica) | 128101442 | 16.75 | 9.37 | 2.94 |
| D24 (second replica) | 146232426 | 16.00 | 8.98 | 2.79 |
| D48 (first replica) | 129063180 | 18.10 | 10.01 | 3.05 |
| D48 (second replica) | 159699192 | 16.65 | 9.50 | 2.90 |

Table S4. Intrapopulation and interpopulation genetic distances of Nigerian populations.

| π | Tashan nabai | Panbalarabe | Wak | Jere | Gishiri | Anguantuta |
| --- | --- | --- | --- | --- | --- | --- |
| Tashan nabai | 0.004 |  |  |  |  |  |
| Panbalarabe | 0.007 | 0.007 |  |  |  |  |
| Wak | 0.005 | 0.008 | 0.005 |  |  |  |
| Jere | 0.012 | 0.013 | 0.013 | 0.004 |  |  |
| Gishiri | 0.013 | 0.014 | 0.014 | 0.005 | 0.004 |  |
| Anguantuta | 0.009 | 0.011 | 0.01 | 0.014 | 0.015 | 0.0057 |

Table S5. Morphological analysis of superior volsella of male genitalia of *P. vanderplanki* populations.

| Population | Analyzed sample size | Observed superior volsella lateral setae | Mean number of observed superior volsella lateral setae | SD |
| --- | --- | --- | --- | --- |
| Wak | 10 | 1 | 0.1 | 0.3 |
| Tashan nabai | 10 | 2 | 0.2 | 0.4 |
| Panbalarabe | 10 | 2 | 0.2 | 0.4 |
| Anguantuta | 10 | 0 | 0 | 0 |
| Jere | 10 | 0 | 0 | 0 |
| Gishiri | 8 | 0 | 0 | 0 |

Table S6. Copy number variation of *PvPIMT* genes

| Gene | Tashan nabai (average coverage) | Estimated copy number | Panbalarabe (average coverage) | Estimated copy number | Jere (average coverage) | Estimated copy number | Gishiri (average coverage) | Estimated copy number | Wak (average coverage) | Estimated copy number | Anguantuta (average coverage) | Estimated copy number | Estimated copy number across population |
| --- | --- | --- | --- | --- | --- | --- | --- | --- | --- | --- | --- | --- | --- |
| PvPIMT1 | 19,4 | 1,61 | 15,73 | 1,1 | 9,63 | 0,94 | 19,23 | 1,28 | 31,56 | 1,84 | 9,62 | 0,93 | 1,28 |
| PvPIMT2 | 12,19 | 1,01 | 23,35 | 1,63 | 7,59 | 0,74 | 22,8 | 1,52 | 17,37 | 1,01 | 11,67 | 1,13 | 1,17 |
| PvPIMT3 | 10,74 | 0,89 | 18,46 | 1,29 | 11 | 1,07 | 18,28 | 1,22 | 15,58 | 0,91 | 7,21 | 0,7 | 1,01 |
| PvPIMT4 | 17,21 | 1,43 | 18,18 | 1,27 | 13,84 | 1,35 | 22,96 | 1,53 | 23,58 | 1,37 | 15,55 | 1,5 | 1,41 |
| PvPIMT5 | 14,57 | 1,21 | 16,93 | 1,18 | 10,99 | 1,07 | 24,53 | 1,64 | 17,22 | 1 | 14,9 | 1,44 | 1,26 |
| PvPIMT6 | 15,06 | 1,25 | 13,83 | 0,97 | 12,09 | 1,18 | 12,21 | 0,82 | 20,87 | 1,22 | 14,09 | 1,36 | 1,13 |
| PvPIMT7 | 16,16 | 1,34 | 19,75 | 1,38 | 12,87 | 1,26 | 15,69 | 1,05 | 20,21 | 1,18 | 15,35 | 1,48 | 1,28 |
| PvPIMT8 | 9,07 | 0,75 | 20,49 | 1,43 | 10,43 | 1,02 | 24,04 | 1,6 | 15,96 | 0,93 | 7,04 | 0,68 | 1,07 |
| PvPIMT9 | 15,7 | 1,31 | 16,07 | 1,12 | 7,23 | 0,71 | 18,79 | 1,25 | 18,43 | 1,07 | 8,73 | 0,84 | 1,05 |
| PvPIMT10 | 9,53 | 0,79 | 15,1 | 1,06 | 13,51 | 1,32 | 20,81 | 1,39 | 13,47 | 0,79 | 11,86 | 1,15 | 1,08 |
| PvPIMT11 | 16,73 | 1,39 | 17,36 | 1,21 | 12,15 | 1,19 | 25,06 | 1,67 | 18,82 | 1,1 | 9,02 | 0,87 | 1,24 |
| PvPIMT12 | 12,55 | 1,04 | 18,69 | 1,31 | 13,02 | 1,27 | 18,62 | 1,24 | 12,85 | 0,75 | 8,51 | 0,82 | 1,07 |
| PvPIMT13 | 14,99 | 1,25 | 15,93 | 1,11 | 8,36 | 0,82 | 20,78 | 1,39 | 13,85 | 0,81 | 16,27 | 1,57 | 1,16 |
| PvPIMT14 | 11,38 | 0,95 | 21,71 | 1,52 | 14,23 | 1,39 | 15,28 | 1,02 | 15,14 | 0,88 | 8,17 | 0,79 | 1,09 |

Table S7. πN/πS ratio of PvPIMT genes in all Nigerian midge populations (except Anguantuta). Asterisks in table S7 mean that πN or πS of this particular PIMT is zero, so the πN/πS of this PIMT cannot be measured.

|  | πN/πS | | | | |
| --- | --- | --- | --- | --- | --- |
| Genes | Tashan nabai | Panbalarabe | Jere | Gishiri | Wak |
| PvPIMT1 | * | 0,05 | * | * | * |
| PvPIMT2 | 0,068 | 0,04 | 0,594 | 0,044 | 0,051 |
| PvPIMT3 | * | 0,019 | * | * | * |
| PvPIMT4 | 0,046 | 0,206 | * | 0,03 | 0,065 |
| PvPIMT5 | 0,035 | 0,087 | 0,06 | 0,049 | 0,074 |
| PvPIMT6 | 0,113 | 0,11 | 0,033 | 0,056 | 0,098 |
| PvPIMT7 | 0,02 | 0,032 | * | * | 0,129 |
| PvPIMT8 | * | 0,372 | 0,06 | 0,395 | 0,545 |
| PvPIMT9 | 0,078 | 0,021 | * | 0,082 | 0,065 |
| PvPIMT10 | * | * | * | 0,02 | * |
| PvPIMT11 | 0,105 | 0,041 | * | 0,024 | 0,037 |
| PvPIMT12 | 0,035 | 0,066 | 0,02 | 0,043 | 0,045 |
| PvPIMT13 | 0,018 | 0,014 | * | 0,043 | 0,037 |
| PvPIMT14 | 0,096 | 0,084 | 0,029 | 0,059 | 0,051 |

Table S8. Genetic distance between *P. vanderplanki* and *P. pembai.* Chikopa is the name of *P. pembai* population

| Populations | π (all genome) | π (chr4) | π (chr3) | π (chr2) | π (chr1) | Mean Fst along full genome |
| --- | --- | --- | --- | --- | --- | --- |
| Tash vs. Chikopa | 0.078 | 0.098 | 0.079 | 0.076 | 0.076 | 0.9 |
| Pan vs. Chikopa | 0.076 | 0.1 | 0.077 | 0.074 | 0.074 | 0.85 |
| Jere vs. Chikopa | 0.08 | 0.1 | 0.081 | 0.078 | 0.078 | 0.91 |
| Gishiri vs. Chikopa | 0.079 | 0.098 | 0.079 | 0.077 | 0.075 | 0.9 |
| Wak vs. Chikopa | 0.077 | 0.096 | 0.077 | 0.075 | 0.076 | 0.88 |
| Anguantuta vs. Chikopa | 0.079 | 0.098 | 0.079 | 0.077 | 0.076 | 0.89 |

Table S9. Standart McDonald-Kreitman test.

|  | dn/ds | | | | | | | pn/ps | | | | | Standart MK | | | | |
| --- | --- | --- | --- | --- | --- | --- | --- | --- | --- | --- | --- | --- | --- | --- | --- | --- | --- |
| GenevsGene | Tashan nabai | Panbalarabe | Wak | Jere | Gishiri | Average dn/ds for populations | Standart variation | Tashan nabai | Panbalarabe | Wak | Jere | Gishiri | dn/ds (Tashan nabai) / pNpS (Panbalarabe) | dN/dS (Panbalarabe) / pNpS (Panbalarabe) | dN/dS (Wak) / pNpS (Wak) | dN/dS (Jere) / pNpS (Jere) | dN/dS (Gishiri) / pNpS (Gishiri) |
| PvPIMT1 vs PpPIMT1 | 0,086 | 0,085 | 0,086 | 0,089 | 0,090 | 0,087 | 0,00204 | * | 0,05 | * | * | * | * | 1,7050248 | * | * | * |
| PvPIMT2 vs PpPIMT2 | 0,141 | 0,132 | 0,147 | 0,139 | 0,142 | 0,140 | 0,00560 | 0,068 | 0,04 | 0,051 | 0,594 | 0,044 | 2,07445 | 3,299812351 | 2,888950226 | 0,2333701757 | 3,231972194 |
| PvPIMT3 vs PpPIMT3-1 | 0,346 | 0,350 | 0,336 | 0,340 | 0,350 | 0,344 | 0,00614 | * | 0,019 | * | * | * | * | 18,43122502 | * | * | * |
| PvPIMT3 vs PpPIMT3-2 | 0,320 | 0,333 | 0,292 | 0,320 | 0,328 | 0,319 | 0,01608 | * | 0,019 | * | * | * | * | 17,53496152 | * | * | * |
| PvPIMT4 vs PpPIMT4-1 | 0,428 | 0,424 | 0,428 | 0,421 | 0,432 | 0,427 | 0,00433 | 0,046 | 0,206 | 0,065 | * | 0,03 | 9,310458895 | 2,058919379 | 6,588940141 | * | 14,39969149 |
| PvPIMT4 vs PpPIMT4-2 | 0,388 | 0,387 | 0,399 | 0,384 | 0,398 | 0,391 | 0,00707 | 0,046 | 0,206 | 0,065 | * | 0,03 | 8,424170542 | 1,877610075 | 6,143040985 | * | 13,28207613 |
| PvPIMT4 vs PpPIMT4-3 | 0,560 | 0,556 | 0,565 | 0,552 | 0,577 | 0,562 | 0,00982 | 0,046 | 0,206 | 0,065 | * | 0,03 | 12,17374271 | 2,700519519 | 8,699994326 | * | 19,24930642 |
| PvPIMT4 vs PpPIMT4-4 | 0,455 | 0,452 | 0,542 | 0,449 | 0,471 | 0,474 | 0,03892 | 0,046 | 0,206 | 0,065 | * | 0,03 | 9,899554827 | 2,195556691 | 8,336414343 | * | 15,71009936 |
| PvPIMT4 vs PpPIMT4-5 | 0,481 | 0,481 | 0,496 | 0,478 | 0,504 | 0,488 | 0,01148 | 0,046 | 0,206 | 0,065 | * | 0,03 | 10,44873207 | 2,334865262 | 7,630248798 | * | 16,79701267 |
| PvPIMT4 vs PpPIMT4-6 | 0,420 | 0,420 | 0,426 | 0,417 | 0,438 | 0,424 | 0,00863 | 0,046 | 0,206 | 0,065 | * | 0,03 | 9,125750187 | 2,03931579 | 6,557120799 | * | 14,61279745 |
| PvPIMT5 vs PpPIMT6 | 0,390 | 0,391 | 0,399 | 0,376 | 0,386 | 0,388 | 0,00843 | 0,035 | 0,087 | 0,074 | 0,06 | 0,049 | 11,14948402 | 4,489879031 | 5,388703506 | 6,261302335 | 7,871688856 |
| PvPIMT6 vs PpPIMT6 | 0,288 | 0,255 | 0,272 | 0,311 | 0,301 | 0,285 | 0,02227 | 0,113 | 0,11 | 0,098 | 0,033 | 0,056 | 2,547664149 | 2,318785838 | 2,77447002 | 9,411993488 | 5,372857914 |
| PvPIMT7 vs PpPIMT6 | 0,267 | 0,286 | 0,268 | 0,255 | 0,266 | 0,268 | 0,01115 | 0,02 | 0,032 | 0,129 | * | * | 13,34114899 | 8,941476371 | 2,079265307 | * | * |
| PvPIMT8 vs PpPIMT7 | 0,283 | 0,271 | 0,303 | 0,277 | 0,313 | 0,289 | 0,01785 | * | 0,372 | 0,545 | 0,06 | 0,395 | * | 0,7282213667 | 0,5563213273 | 4,622773063 | 0,7922726883 |
| PvPIMT9 vs PpPIMT8 | 0,780 | 0,800 | 0,749 | 0,766 | 0,298 | 0,678 | 0,21371 | 0,078 | 0,021 | 0,065 | * | 0,082 | 9,998805714 | 38,07734407 | 11,5300861 | * | 3,62862413 |
| PvPIMT9 vs PpPIMT9 | 0,226 | 0,226 | 0,249 | 0,195 | 0,230 | 0,225 | 0,01937 | 0,078 | 0,021 | 0,065 | * | 0,082 | 2,89234452 | 10,76596133 | 3,830609398 | * | 2,807876745 |
| PvPIMT10 vs PpPIMT10 | 0,123 | 0,121 | 0,117 | 0,134 | 0,134 | 0,126 | 0,00741 | * | * | * | * | 0,02 | * | * | * | * | 6,677011644 |
| PvPIMT11 vs PpPIMT11 | 0,253 | 0,263 | 0,256 | 0,256 | 0,242 | 0,254 | 0,00777 | 0,105 | 0,041 | 0,037 | * | 0,024 | 2,408485606 | 6,426169648 | 6,920549443 | * | 10,08707709 |
| PvPIMT12 vs PpPIMT12 | 0,315 | 0,319 | 0,319 | 0,319 | 0,309 | 0,316 | 0,00453 | 0,035 | 0,066 | 0,045 | 0,02 | 0,043 | 8,993772371 | 4,83895847 | 7,078430142 | 15,96856295 | 7,181668951 |
| PvPIMT13 vs PpPIMT12 | 0,480 | 0,441 | 0,453 | 0,473 | 0,425 | 0,454 | 0,02277 | 0,018 | 0,014 | 0,037 | * | 0,043 | 26,66819434 | 31,4920091 | 12,23798987 | * | 9,875064726 |
| PvPIMT14 vs PpPIMT13 | 0,226 | 0,217 | 0,221 | 0,241 | 0,231 | 0,227 | 0,00952 | 0,096 | 0,084 | 0,051 | 0,029 | 0,059 | 2,356082424 | 2,581536467 | 4,328632818 | 8,323704923 | 3,909308891 |
| PvPIMT14 vs PpPIMT14 | 0,205 | 0,203 | 0,198 | 0,206 | 0,204 | 0,203 | 0,00339 | 0,096 | 0,084 | 0,051 | 0,029 | 0,059 | 2,131399515 | 2,417892957 | 3,872562788 | 7,118105306 | 3,462388608 |

*not applicable

Table S10. Variant calling statistics after filtration.

| **Population name** | **Tashan nabai** | **Panbalarabe** | **Wak** | **Anguantuta** | **Jere** | **Gishiri** | **Chikopa (Malawi)** |
| --- | --- | --- | --- | --- | --- | --- | --- |
| **Number of variants** | 1,024,4  36 | 1,808,169 | 1,686,913 | 1,512,192 | 1,328,7  54 | 2,019,494 | 1,422,879 |
| **Number of multi- allelic variants** | 4,594 | 5,985 | 8,843 | 4,661 | 11,940 | 20,405 | 2,048 |
| **SNPs** | 942,308 | 1,704,441 | 1,543,897 | 1,357,140 | 1,212,9  69 | 1,824,780 | 1,294,544 |
| **Insertions** | 46,107 | 59,602 | 80,030 | 80,420 | 66,361 | 112,396 | 60,535 |
| **Deletions** | 40,618 | 50,117 | 71,835 | 74,632 | 61,364 | 102,723 | 67,800 |
| **Nonsynonymous variants** | 34.24% | 34.03% | 37.08% | 38.41% | 32.36% | 34.28% | 40.80% |
| **Synonymous variants** | 65.50% | 65.70% | 62.59% | 61.17% | 67.40% | 65.46% | 58.76% |
| **Nonsense variants** | 0.25% | 0.27% | 0.32% | 0.42% | 0.23% | 0.25% | 0.45% |

Table S11. Precise coordinate of sample populations of *P. vanderplanki.*

| Population | Type | lat_dec | lon_dec |
| --- | --- | --- | --- |
| Tashan nabai | north | 11.35888 | 7.944721 |
| Panbalarabe | north | 11.090905 | 7.656434 |
| Wak | north | 11.602667 | 8.356316 |
| Gishiri | south | 9.431507 | 7.397349 |
| Jere | south | 9.569375 | 7.43143 |
| Anguantuta | south | 10.246236 | 7.341894 |
